# Priming effects on labile and stable soil organic carbon decomposition: Pulse dynamics over two years

**DOI:** 10.1371/journal.pone.0184978

**Published:** 2017-09-21

**Authors:** Xiuwei Zhang, Xiaozeng Han, Wantai Yu, Peng Wang, Weixin Cheng

**Affiliations:** 1 Key Laboratory of Forest Ecology and Management, Institute of Applied Ecology, Chinese Academy of Sciences, Shenyang, China; 2 University of Chinese Academy of Sciences, Beijing, China; 3 Northeast Institute of Geography and Agroecology, Chinese Academy of Sciences, Harbin, China; 4 Environmental Studies Department, University of California, Santa Cruz, California, United States of America; Pacific Northwest National Laboratory, UNITED STATES

## Abstract

Soil organic carbon (SOC) is a major component in the global carbon cycle. Yet how input of plant litter may influence the loss of SOC through a phenomenon called priming effect remains highly uncertain. Most published results about the priming effect came from short-term investigations for a few weeks or at the most for a few months in duration. The priming effect has not been studied at the annual time scale. In this study for 815 days, we investigated the priming effect of added maize leaves on SOC decomposition of two soil types and two treatments (bare fallow for 23 years, and adjacent old-field, represent stable and relatively labile SOC, respectively) of SOC stabilities within each soil type, using a natural ^13^C-isotope method. Results showed that the variation of the priming effect through time had three distinctive phases for all soils: (1) a strong negative priming phase during the first period (≈0–90 days); (2) a pulse of positive priming phase in the middle (≈70–160 and 140–350 days for soils from Hailun and Shenyang stations, respectively); and (3) a relatively stabilized phase of priming during the last stage of the incubation (>160 days and >350 days for soils from Hailun and Shenyang stations, respectively). Because of major differences in soil properties, the two soil types produced different cumulative priming effects at the end of the experiment, a positive priming effect of 3–7% for the Mollisol and a negative priming effect of 4–8% for the Alfisol. Although soil types and measurement times modulated most of the variability of the priming effect, relative SOC stabilities also influenced the priming effect for a particular soil type and at a particular dynamic phase. The stable SOC from the bare fallow treatment tended to produce a narrower variability during the first phase of negative priming and also during the second phase of positive priming. Averaged over the entire experiment, the stable SOC (i.e., the bare fallow) was at least as responsive to priming as the relatively labile SOC (i.e., the old-field) if not more responsive. The annual time scale of our experiment allowed us to demonstrate the three distinctive phases of the priming effect. Our results highlight the importance of studying the priming effect by investigating the temporal dynamics over longer time scales.

## Introduction

Global soil organic carbon (SOC) stock is largely regulated by plant input from primary production and the release of CO_2_ from microbial decomposition of SOC [1]. The input can also influence the output (i.e., CO_2_ release) through a phenomenon called “priming effect”, which was first discovered by Löhnis (1926) [2] and was commonly defined as the stimulation or suppression of SOC decomposition by fresh input of organic substrates such as plant litter [3].

The input of fresh organic matter can either maintain the existing SOC level [3] or enhance the decomposition of stabilized SOC, which may persist long after the exhaustion of the added fresh organic matter, ultimately resulting in net soil carbon loss [4–5]. The role of the priming effect in regulating soil carbon balance has recently become a focal area of research [6]. Although the potential impact of the priming effect on SOC dynamics is widely recognized [7], incorporating the priming effect into global carbon models remains challenging, mostly due to the large uncertainties associated with both the direction and the magnitude of measured priming effects [8–9]. Not knowing which components of the entire SOC stock are vulnerable to priming can be a major cause of these uncertainties [10]. Therefore, a crucial research question is: does the priming effect impact the stable SOC fraction more than the labile SOC fraction?

Soil organic carbon commonly exists as a mixture of many heterogeneous components with different physiochemical properties, stabilities, and turnover times [11]. Based on their turnover times, SOC stocks have been conveniently divided into three components: (1) annually cycling, labile SOC, (2) decadally cycling stable SOC, and (3) millennially cycling, largely inert SOC; each contributing to approximately 0–5%, 60–85%, and 10–40% of total SOC stocks, respectively [1]. Labile SOC is mainly consisted of microbial biomass and recent inputs (e. g., plant litter and root exudates), which would not contribute to major changes in total SOC because of its much smaller size and very fast turnover. Millennially cycling SOC is recently identified as mostly consisted of black carbon or other largely inert materials [12]. More importantly, decadally cycling stable SOC with turnover times of 10–100 years is the dominant component of SOC stocks, and how it may response to the priming effect will significantly impact the global C cycle [13–14]. Contrasting topsoils with subsoils has been used to study the priming effect on different SOC stocks, because SOC in subsoils is commonly regarded as mainly consisted of stable forms. The interactive effects of input quality and quantity on priming of topsoils and subsoils have been illustrated in some recent studies [15–16], and often find inconsistent results between topsoils and subsoils. Positive priming effects tend to be more pronounced in soils that contain SOC of relatively low biodegradability [17–18]. These results tend to support the hypothesis that fresh organic matter input promotes stable SOC decomposition through alleviating energy limitation and stimulating microbial activities. With more energy available, microbial activities increase, producing more extracellular enzymes which subsequently enhance SOC decomposition [19]. However, it also depends upon the nature of organic material: labile substrates generally produce less priming effect, in contrast, relatively recalcitrant substrates often result in higher priming via co-metabolism [3, 20]. However, the above-mentioned hypothesis is not supported by evidence from some other experiments. For example, fructose addition only produced significant priming effect in the topsoil but not in the subsoil that contained mostly stable SOC [15]. The following three aspects may have contributed to this inconsistency. First, the experimental durations are often too short for assessing the dynamic response of stabilized SOC to priming. This is often due to method limitations which are more severe at longer durations when rates of SOC decomposition decline to a fraction of the rates at early periods while measurement errors remain largely the same [14]. Second, measurements of the priming effect by using cumulative CO_2_ evolution often obscure the pulse-dynamic nature of the priming effect [21]. Third, glucose, often utilized for studying priming effects, may not be suitable for investigating the response of stabilized SOC to priming [22–23]. In this study, we aim to address these potential issues.

In this study, we differentiated SOC components by using soils from two long-term bare fallow plots and soils from adjacent abandoned farm plots. The two bare fallow soils were deprived of fresh carbon inputs for 23 years, which largely represent stable SOC. The adjacent old-field soils received continuous fresh organic matter input, which represent labile SOC in the context of our study. Using a dynamic CO_2_ trapping system, we carried out an incubation experiment for 815 days. The improved measuring system allowed us to reliably monitor the decomposition rates for both the fresh organic matter and the stabilized SOC. Maize leaves were used as the fresh organic matter input for assessing the priming effect. The dynamic CO_2_ trapping system substantially reduced experimental errors, especially during the later period of the incubation. The main objectives of this study were (i) to investigate whether the priming effect differs between stable SOC (using the bare fallow soil) and labile SOC (using the old-field soil), and (ii) to investigate the dynamic changes of priming effects over a longer period of time (815 days).

## Material and methods

### Location

We selected two experimental stations of Chinese Academy of Sciences (China): Hailun Agricultural experimental station (47°26′N, 126°38′E) in Heilongjiang province and Shenyang Station (41°32′N, 122°23′E) in Liaoning province in Northeastern China (S1 Fig). Both sites have typical continental monsoon climate. At Hailun Station, the mean annual temperature is 1.5°C, and the mean annual precipitation is 550 mm (Table 1). The soil at Hailun Station was classified as an Aquic Mollisol with a deep A-horizon and predominantly Montmorillonite clay. The old-field and the bare fallow treatments were applied onto a crop field which had been cultivated with row crops (C_3_ crops) for approximately 60 years without any applications of synthetic fertilizers before the experiment. The old-field treatment cultivation stopped in 1985. At the time of the soil sampling for the current study, the vegetation in the old-field treatment has become a mesic natural meadow with the dominant plant groups in the Poaceae and the Cyperaceae. At the time of soil sampling, the annual aboveground plant biomass production in the old-field treatment has been approximately 700 g m^-2^.

**Table 1:** Characteristics of the soils and the maize leaves used in the experiment. Different letters indicate significant (P<0.05) differences in soil properties between old-field and bare fallow soils from the same site.

Sites/treatments	Hailun			Shenyang		Maize leaves
	Old-field		Bare fallow	Old-field	Bare fallow	
**Soil type**	Mollisol		Mollisol	Alfisol	Alfisol	
**Total carbon (mg g**^**-1**^**)**	35.55a		21.81b	16.60a	11.44b	379.00
**Total N (mg g**^**-1**^**)**	2.65a		1.78b	1.37a	0.94b	13.37
**C/N**	13.45a		12.26b	12.08a	12.17a	28.27
**pH (1:2.0 soil: water)**	6.17a		6.01a	6.01a	6.10a	─
**Sand (%) (2–0.05 mm)**	12.30a		11.49a	26.41a	21.95a	─
**Silt (%) (0.05–0.002 mm)**	52.51a		54.74a	60.98a	67.92a	─
**Clay (%) (<0.002 mm)**	35.19a		33.77a	12.61a	10.13a	─
**Initial δ**^**13**^**C of SOC**	-24.76b		-23.99a	-24.22a	-24.00a	─
**δ**^**13**^**C of maize leaves**	─		─	─	─	-13.35

At Shenyang station, the mean annual temperature is 7.5°C, and the mean annual precipitation is 690 mm. The soil at Shenyang Station was classified as an Aquic Alfisol developed from silty sediments. The old-field and the bare fallow treatments were established on a ceased rice paddy field. Secondary plant succession in the old-field treatment at Shenyang station started in 1990 and has become a meadow mixed with common reed (*Phragmites australis*). At the time of soil sampling, the annual aboveground plant biomass production in the old-field treatment has been approximately 600 g m^-2^. As part of the coordinated research network, the bare fallow plots at both stations have been kept free of vegetation by frequent hand weeding and annually plowing for 23 years at the time of soil sampling for this study.

### Soil sampling

Soils were sampled from the plow layer (0~20 cm) in the fields under long-term bare fallow treatment and the adjacent old-field treatment. Within each treatment, soils were sub-sampled at four randomly selected sites and homogenized into one sample. Prior to the incubation experiment, all soils were sieved through 2 mm screen; Fine roots, other plant debris and visible stones were removed carefully by handpicking and electrostatic method. All soil samples were homogenized, air-dried, and stored at 20°C before incubation.

### Experimental design and addition of maize leaves

We took the advantage of the soils from two long-term experimental manipulations at Hailun and Shenyang Station. The bare fallow and old-field treatments in these two stations would help us to decipher the vulnerability to the priming effect between labile and stabilized SOC. The soils under the treatment of bare fallow for 23 years at both sites were used to represent the case of relatively stabilized SOC as the main source for decomposition, while the soils under the old-field treatment was used to represent the case of predominantly labile SOC as the main source for decomposition. Therefore, we referred the bare fallow SOC as “stable” and the old-field SOC as “labile” in this study.

For each of the four soils (Table 1), three replicates of sub-samples were prepared as the no-amendment controls, each weighed 200 g dry weight and was placed in a plastic container made of PPR plastic pipe (20 cm in length, 5.2 cm in diameter). In the substrate-amended treatment, 6 grams of ground maize leaves were mixed into 200 g of dry soil, and paced into a plastic container (the same kind as in the control). Based on commonly reported decomposition rates of maize leaves [24–25], this amount of maize leaves added in the amendment was considered necessary to produce a measurable ^13^C-signal (i.e., a C_4_ signal) throughout the incubation period. Three replicates were also used for the amended treatment. The maize leaves were ground into pieces <2 mm in length using an automatic pulverizer (CS-700, COSUAI, Wuyi hainer electric appliance co. LTD, Zhejiang, China) before mixing into the soil. The moisture content in each incubation was carefully maintained at 60% of the water holding capacity by frequent weighing and watering with deionized water. Each plastic container was closed with one-hole rubber stopper connected to ventilation tubing and incubated in processor-controlled incubators (SHELLAB LI20-2, Sheldon Manufacturing Inc., Cornelius, OR, USA, with a temperature control accuracy and evenness of ±0.02°C) at 20°C for 815 days. Three identical incubators were used as true replicates, each incubator contained a replicate set of plastic containers from individual treatments. A continuous aerobic condition in each plastic container was maintained via automatic timer-controlled aeration with fresh air for one hour in each 4-hours interval during the entire incubation experiment.

### Soil respiration and δ^13^C-CO_2_

**Fig 1:**
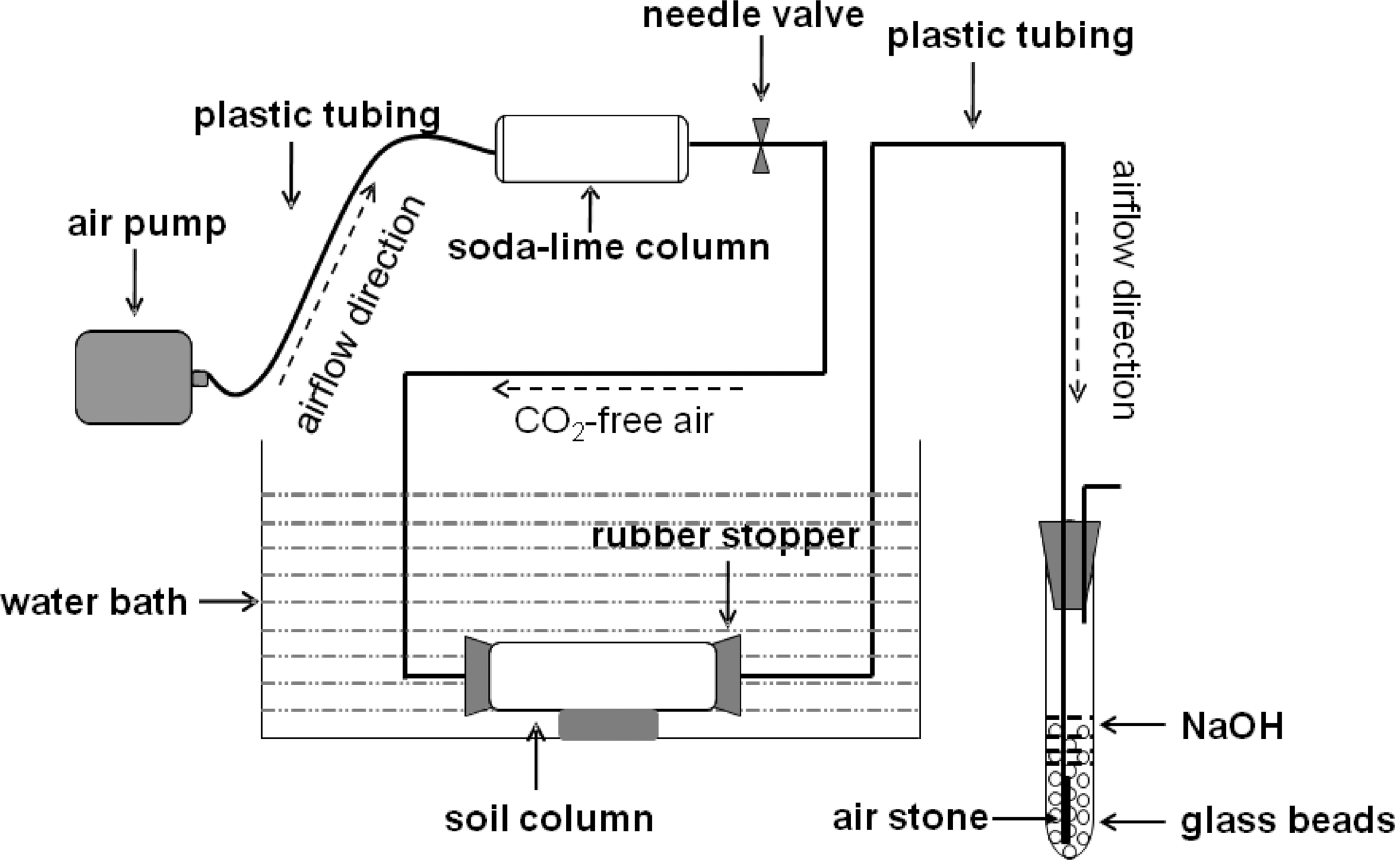
The dynamic CO_2_ trapping system for accurately measuring soil respiration and δ^13^C of respired CO_2_.

Soil respiration rate was measured using an improved dynamic CO_2_ trapping system (Fig 1). Briefly, an air pump forces ambient air through a soda-lime column, thereby producing 100% CO_2_-free air (checked by using an infrared CO_2_ analyzer, LiCOR 6262, Lincoln, NB, USA). The CO_2_-free air then entered the manifold, from which individual tubing leaded to individual sample inflow tubing. A needle valve was used to control the air flow to each sample in order to ensure a constant flow rate (the average flow rate for all samples was at 60–65 mL min^-1^). One of the outlets was connected to an empty plastic container and was used as a blank reference. After an one-hour stabilizing/equilibrating period, respiration measurements were initiated by connecting the outflow tubing of each sample to the CO_2_ trapping glass tube (length = 20 cm, diameter = 1.8 cm) containing 12 mL of 0.5 M NaOH solution and glass beads. A glass airstone was connected to the end of the air-outlet tubing submerged in the NaOH solution, so that small air bubbles were produced at the bottom of the CO_2_ trapping glass tube and passed through the trapping solution. At each measurement time, CO_2_ released from soil respiration was trapped in a 12 mL 0.5 M NaOH solution for one hour at every 6-hour intervals during an entire 24 hour or longer (if needed) period. The CO_2_ trapping efficiency of this system was greater than 99%. The CO_2_ trapping was applied at day-8, 14, 28, 45, 60, 75, 90, 128, 144, 158, 187, 202, 240, 263, 299,330,360, 410, 480, 550, 585, 630, 670, 702, 756, 815, respectively. After CO_2_ trapping, the NaOH solution was directly analyzed for total inorganic carbon using multi N/C® 2000 (Analytik Jena, Germany) and δ^13^C using cavity ring-down spectroscopy (CRDS) coupled with an Automate Module (Picarro G2131-i Analyzer, Picarro Inc., Santa Clara, CA, USA), which converts CO_3_^2-^ in the NaOH solution to pure CO_2_ gas before entering into the Picarro ^13^C analyzer. Blanks without soil were included to quantify the amount of C in the blank NaOH solution and C-CO_2_ absorbed during sample handling and processing. The effect of the contaminant C in the blanks on the δ^13^C value of each sample was corrected according to Cheng et al. [26]. Using the natural ^13^C-isotope method, we can partition the SOC- (C_3_ source) and maize- (C_4_ source) derived carbon within total CO_2_. This measurement system allowed us to accurately measure both soil respiration rates and δ^13^C-CO_2_ values throughout the entire experimental period of 815 days.

### Soil microbial biomass carbon

Soil microbial biomass carbon (MBC) was determined using the chloroform fumigation extraction method [27] with some modifications. We did the destructive soil sampling at day-8, 14, 28, 45, 60, 90, 128, 158, 202, 240, 263, 330, 410, 480, 550, 630, 670, 702 and 815. At each destructive sampling time, soil samples in plastic container were carefully homogenized. Paired 40 g soil samples were either extracted with 0.05 M K_2_SO_4_ [28] in a 1:2 ratio or fumigated with chloroform for 24 h (protected against exposure to light) and then extracted in the same way. The extract was analyzed for total organic carbon using multi N/C® 2000 (Analytik Jena, Germany). MBC was calculated from the difference of K_2_SO_4_-extractable C between the fumigated and the non-fumigated soil samples using a k_EC_ factor of 0.38 [29].

### Calculation and statistical analysis

To calculate the contribution of C_3_-C (soil C originated from all C_3_-vegetation) to the total CO_2_ efflux, the following equation was used [30]:

$$C_{3}=C_{t}(\delta_{t}-\delta_{4})/(\delta_{3}-\delta_{4})$$ (1)

Where C_t_ (*C*_*t*_ = *C*_3_ + *C*_4_) was the total carbon of CO_2_ efflux from the substrate amended soil, C_3_ was the C-CO_2_ derived from SOC (the C_3_ source), C_4_ was the C-CO_2_ derived from the C_4_ maize leaves (C_4_ = C_*t*_ − C_3_), δ_t_ (measured at each sampling time) was the δ^13^C value of the total C-CO_2_ (C_t_), δ_3_ (measured at each sampling time) was the δ^13^C value of the SOC-derived C-CO_2_ from each control soils without maize leaves, δ_4_ was the δ^13^C value of the C_4_ maize leaves (Table 1).

The priming effect (PE) was calculated as the difference between the amount of SOC decomposed in the maize-leave amended treatment and in the associated control [7]:

PE=α×Qsample−Qcontrol (2)

Where Q is the CO_2_ respired from sample or control in μg C-CO_2_ g^-1^ soil d^-1^, and α = (δ_*t*_ − δ_4_)/(δ_3_ − δ_4_).

The relative intensity of priming (PE%) was estimated as a percentage of changes relative to the control CO_2_ production by the following equation [31]:

PE=[(α×Qsample−Qcontrol)/Qcontrol]×100 (3)

For reasonable comparisons among treatments, the soil respiration rate and the priming effect were expressed as μg CO_2_-C per 1 gram of the soil per unit of time. The statistical differences in soil respiration rates and the priming effect between soil types and treatments were analyzed using a two-tailed independent-samples t-test. Analysis of variance (ANOVA) of cumulative priming effect was performed using the General Linear Model with all possible interacting terms. Statistical analyses of all data were performed using SPSS Statistics 20. Differences were considered statistically significant when *P*<0.05.

## Results

### The properties of old-field and bare fallow soils

The organic carbon contents of bare fallow soils were 38.6% and 31.1% lower than the adjacent old-field soils from Hailun and Shenyang, respectively (Table 1). A similar level of decline in total soil nitrogen content also occurred under the treatment of bare fallow (32.8% at Hailun and 31.4% at Shenyang). As the bare fallow soil did not receive any new carbon input during the last 23 years, the soil carbon age should be significantly older than that in the old-field soil. Soil pH values were not significantly changed by the two treatments. The clay content in Hailun soil was significantly higher than that in Shenyang soil. Meanwhile, there were no significant differences of clay, silt and sand contents (%) between the old-field and the bare fallow treatments at both experiment stations. Interestingly, the δ^13^C value of the total SOC under the bare fallow treatment was less negative (or higher ^13^C abundance) than that under the old-field treatment, especially for soils from Hailun Station which was statistically significant (*P*<0.05).

### Dynamics of CO_2_ efflux through time

**Fig 2:**
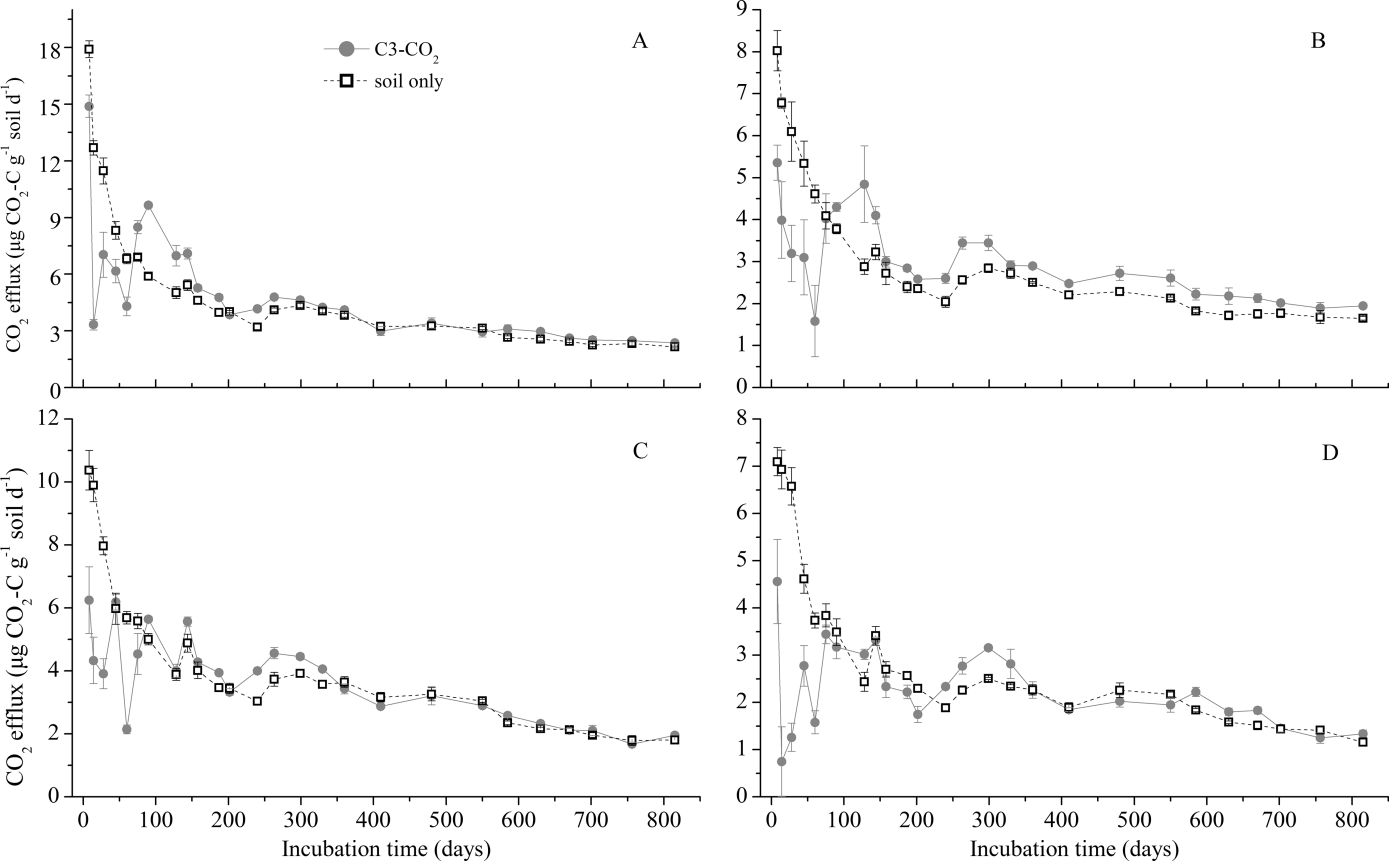
**CO**_**2**_ **efflux rate (μg CO**_**2**_**-C g**^**-1**^ **soil d**^**-1**^**) from SOC mineralization with (circle symbol: C**_**3**_**-CO**_**2**_**) or without (square symbol: soil only) maize leaves amendment for soils taken from the old-field treatment at Hailun Station (A), from the long-term bare fallow treatment at Hailun Station (B); from the old-field treatment at Shenyang Station (C), and from the long-term bare fallow treatment at Shenyang Station (D).** Error bars show ±1 SE.

**Fig 3:**
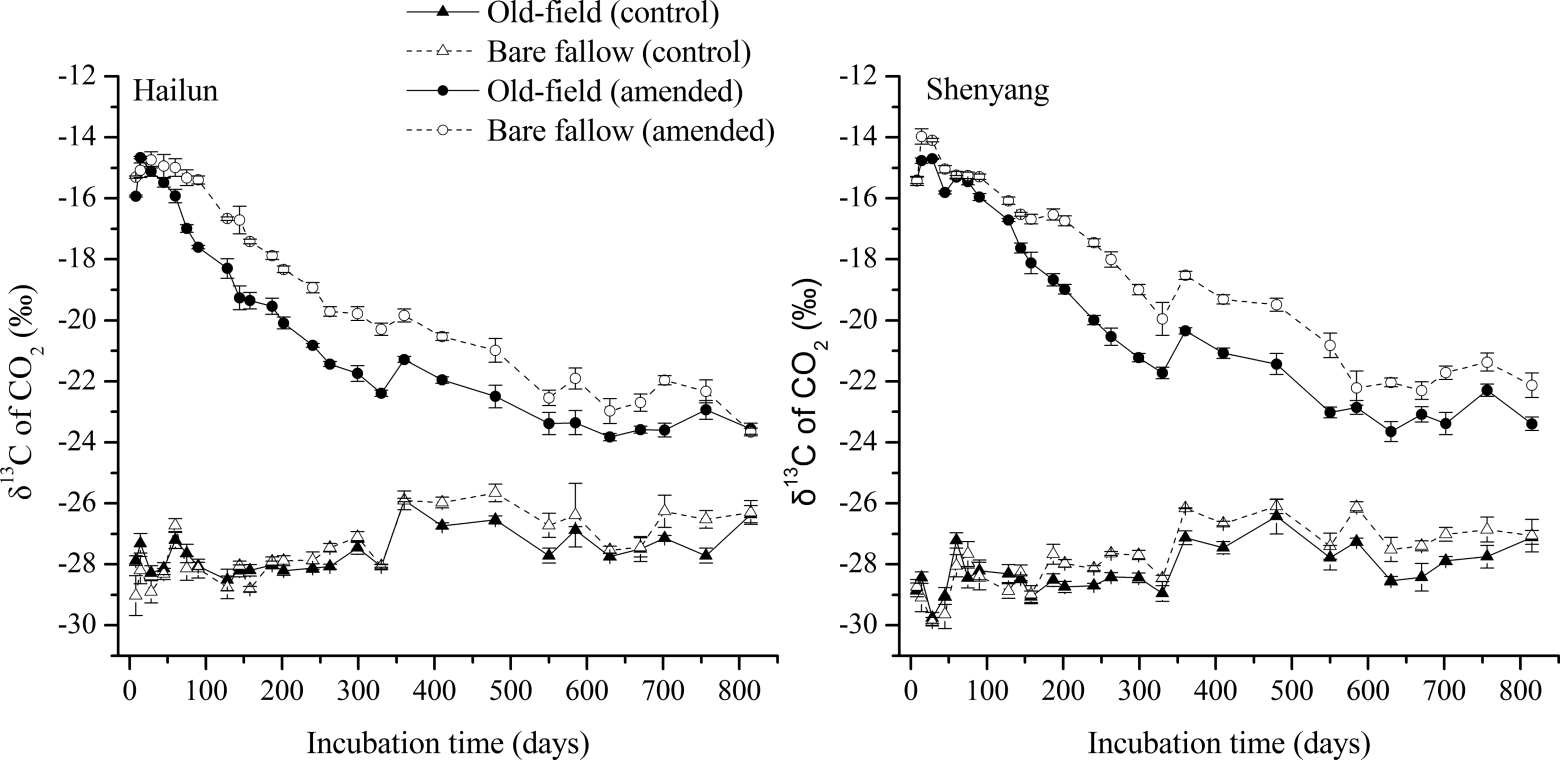
Variation in δ^13^C values of respired CO_2_-C for soils with or without maize leaves amendment taken from old-field and bare fallow treatment at Hailun (left) and Shenyang (right) station throughout the 815-day incubation. Error bars show ±1 SE.

In general, CO_2_ efflux rates from all treatments quickly declined during the initial 200 days of incubation, and tended to stabilize afterwards (Fig 2). The total CO_2_ efflux rate in the treatment with added maize leaves was partitioned into CO_2_ derived from SOC (i.e., C_3_-CO_2_) and CO_2_ derived from maize leaves (i.e., C_4_-CO_2_) using the ^13^C natural tracer method (Eq 1). The δ^13^C-CO_2_ values from treatments amended with maize leaves were high, or near the δ^13^C values of the maize leaves (-13.35‰) at the beginning, and continuously declined to the end of the experiment (Fig 3). This indicated that the proportion of CO_2_ derived from the added maize leaves declined as the time of incubation increased. The δ^13^C-CO_2_ values from the bare fallow soils at both sites were significantly greater than the old-field soils except for the short period at the start of the incubation. This pattern was expected because (1) the δ^13^C values of SOC in the bare fallow soils was greater than that in the old-field soils (Table 1); and (2) relative to the total amounts of SOC, the amount of maize carbon with higher δ^13^C values (-13.35‰) added to the bare fallow soils made up higher proportion than the same amount added to the old-field soils which had higher SOC contents than the bare fallow soils.

**Fig 4:**
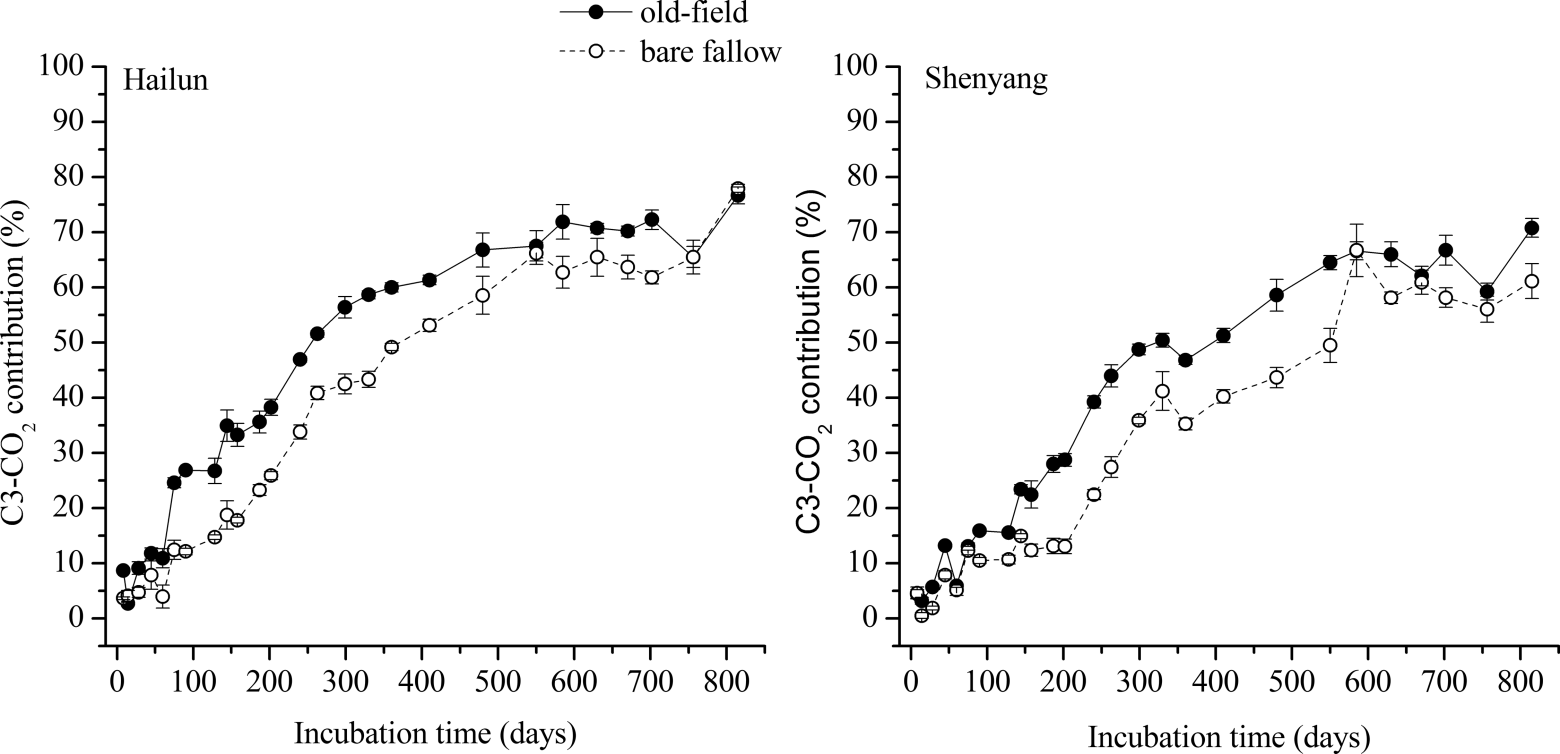
The contribution (%) of SOC-derived CO_2_ to total decomposition of old-field and bare fallow soils from Hailun (left) and Shenyang (right) station when amended with maize leaves. Error bars show ±1 SE.

The relative contribution of CO_2_ production originated from SOC as compared to CO_2_ production originated from maize leaves increased from less than 10% at the start of the experiment to approximately 60–78% as the incubation time increased (Fig 4). At the end of the incubation (815 days), CO_2_ production from SOC decomposition contributed 76.7–77.9% for soils from Hailun Station, and 61.1–70.8% for soils from Shenyang Station. The relative contribution of CO_2_ production originated from SOC of the bare fallow soils was significantly lower than that of the old-field soils from both sites except for the beginning and the end periods when the percent contribution was similar.

In all soils the decomposition rate of the added maize leaves (as the fresh substrate to study the priming effect) was very fast at the start of the incubation, declined rapidly during the initial 70–90 days, showed a noticeable small peak during the period of 90–200 days, then slowly declined to relatively stable rates (S2 Fig).

### Priming effect

**Fig 5:**
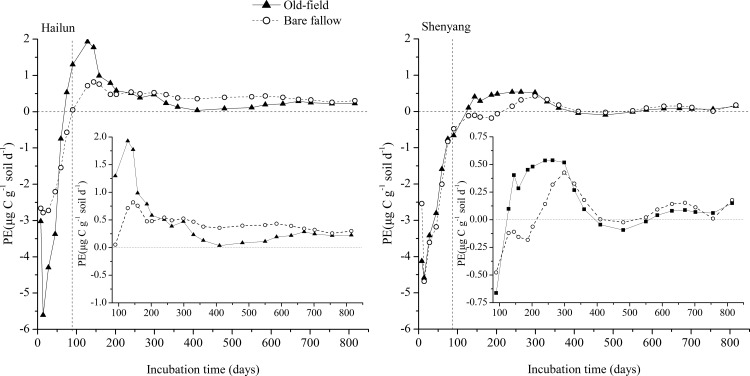
PE (priming effects, instantaneous rate, μg C g^-1^ soil d^-1^) of added maize leaves on SOC decomposition of soils from Hailun (left) and Shenyang (right) station. The inserts show the priming effect starting at day-90 when most treatments started to show a positive priming effect. The running average was obtained using Adjacent-Average method (OriginPro 8.5).

**Fig 6:**
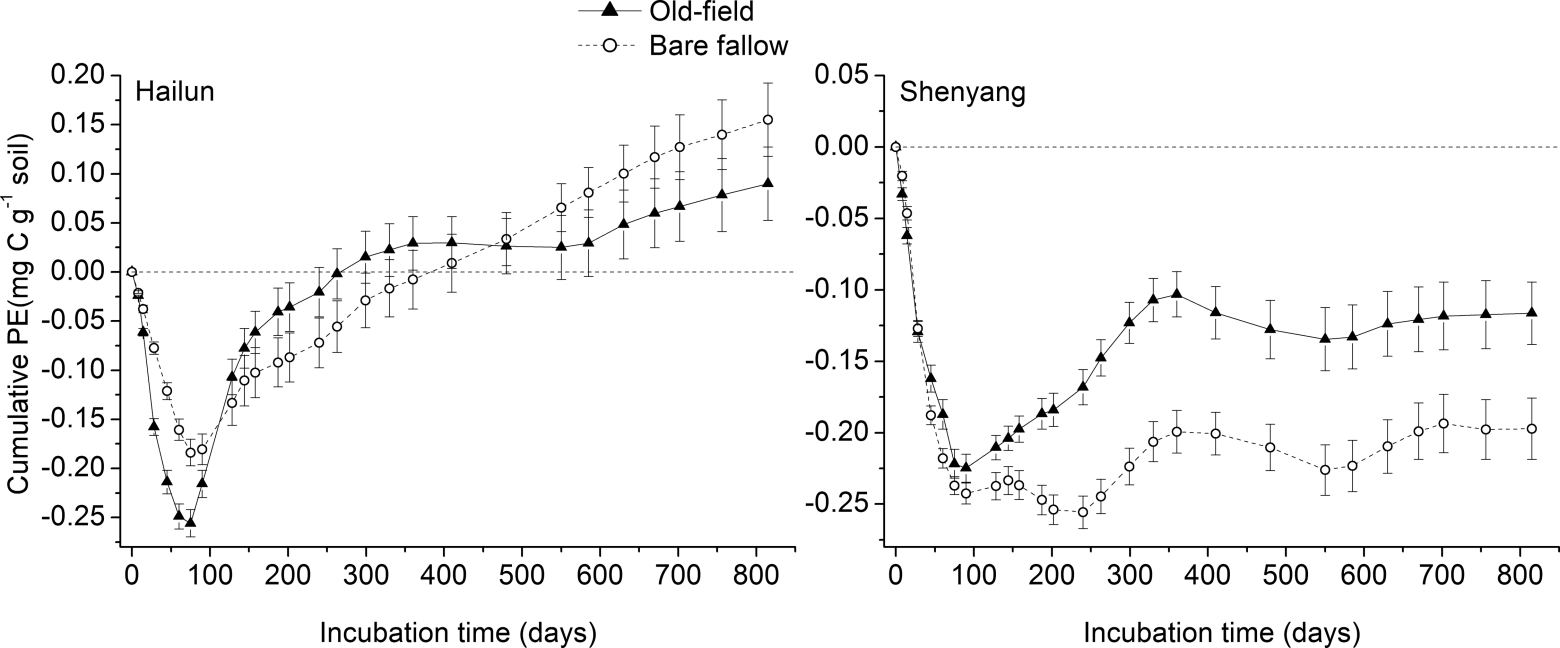
Cumulative PE (priming effects, mg C g^-1^ soil) of added maize on SOC decomposition of old-field (triangle and solid line) and bare fallow (circle and dotted line) soils from Hailun (left) and Shenyang (right) station during 815 days incubation. Error bars show ±1 SE.

The priming effect varied significantly between SOC stabilities, soil type, and incubation time (Fig 5, S1 Table). During the initial period after maize leaves amendment, only a negative priming effect was observed in the four soils. This negative priming effect lasted for 60–75 days in soils from Hailun and 75–90 days in soils from Shenyang (Fig 6). After this period, positive priming effect occurred in all four soils with different durations and intensities. For both the old-field and bare fallow soils from Hailun Station, the positive priming effect was primarily above the zero line (Fig 5). For the two soils from Shenyang station, positive priming effect was less frequently observed compared to Hailun soils. The priming effect in both sites was generally higher in old-field soils than in bare fallow soils during the early incubation period (1–200 days), and the situation switched during the mid- to late stages (Fig 5).

**Fig 7:**
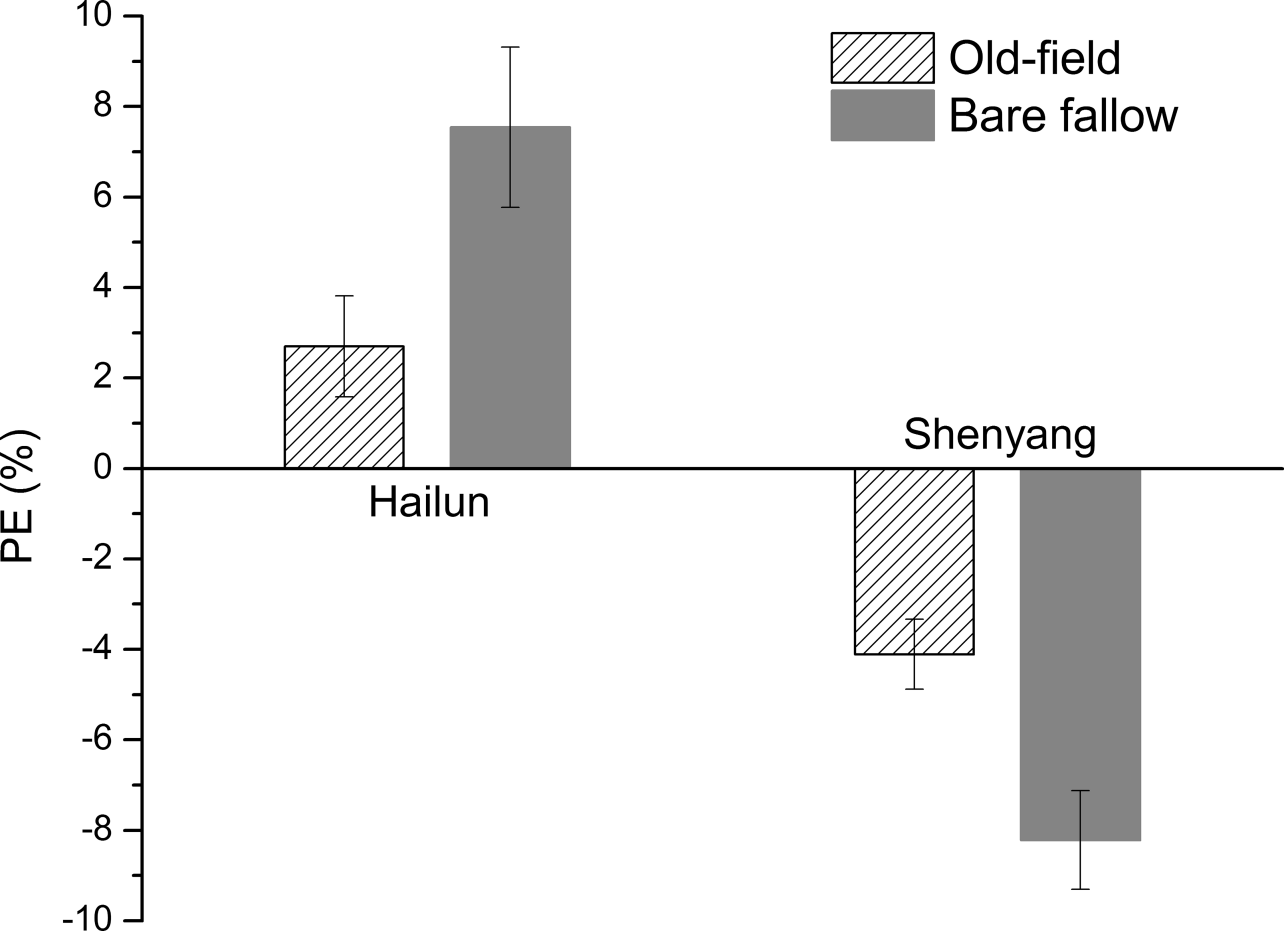
The relative priming effect (PE%, PE% = [(α × Qsample – Qcontrol) / Qcontrol] × 100, where Q is the CO_2_ respired from sample or control, and α is the percentage of SOC-derived CO_2_ to total respiration (%) of old-field and bare fallow soils from Hailun and Shenyang Station after 815 days incubation. Error bars show ±1 SE.

When cumulative CO_2_ evolution rates were used to calculate the priming effect, the soils from the bare fallow treatment produced similar (Shenyang station) or less (Hailun station) negative priming effect during the early incubation period (≈ 45 to 120 days) (Fig 6). However, after the initial period, the soil from the bare fallow treatment at Hailun station produced significantly more negative priming effect than the counterpart old-field soil during the period of 120–460 days, but more positive priming effect during 460–815 days of incubation. The bare fallow soil from Shenyang Station produced a greater negative priming effect than the counterpart old-field soil after the initial period. At the end of the experiment, significantly greater cumulative loss of SOC (positive priming) occurred in both the bare fallow (2.27 and 2.11 mg C g^-1^ soil in maize leaves treated and control soils, respectively) and old-field (3.42 and 3.33 mg C g^-1^ soil in maize leaves treated and control soils, respectively) soils from Hailun station, and significantly less cumulative loss of SOC (negative priming) occurred to both the bare fallow (1.77 and 1.95 mg C g^-1^ soil in maize leaves treated and control soils, respectively) and old-field (2.27 and 2.83 mg C g^-1^ soil in maize leaves treated and control soils, respectively) from Shenyang station, due to the effect of the amendment with maize leaves (S1 Table). For the two treatments from Hailun station, the cumulative priming effect was significantly higher in the bare fallow soil (0.155 mg C g^-1^ soil) than in the old-field soil (0.090 mg C g^-1^ soil). For soils from Shenyang station, the cumulative priming effect was -0.197 and -0.116 mg C g^-1^ soil in the bare fallow soil and in the old-field soil, respectively (Fig 6). When the cumulative priming effect was expressed as a percentage of the cumulative CO_2_ efflux from the control soil without added maize leaves, the bare fallow soil and the old-field soil from Hailun station produced 7.55% and 2.70% positive priming effect, respectively; whereas the negative priming effect was 8.22% and 4.11% for the bare fallow soil and the old-field soil from Shenyang station, respectively (Fig 7).

### Soil carbon balance

At the end of incubation time (815 days), approximately 78–83% of the added maize leaves were mineralized to CO_2_ across all soils amended with maize leaves (S2 Table). Slightly more maize leaves were mineralized in soils from Shenyang station than soils from Hailun station (83% vs. 79% on average). For the total cumulative CO_2_ evolved during the entire experiment, 72% came from the decomposition of maize leaves and 28% from SOC for the old-field soil, while 80% came from maize leaves and 20% from SOC for the bare fallow soil from Hailun station. For soils from Shenyang station, 77% the total cumulative CO_2_ came from maize leaves and 23% from SOC for the old-field soil, and 84% came from maize leaves and 16% from SOC for the bare fallow soil. These high percentages of total CO_2_ from maize leaves were mostly due to the high decomposition rates during the early period of the incubation (S2 Fig).

Over the entire experiment, addition of maize leaves increased the SOC decomposition for soils from Hailun resulting in a positive priming effect, but decreased the SOC decomposition for soils from Shenyang (a negative priming effect) (Fig 7, S2 Table). Compared to the cumulative CO_2_ evolved from the control treatment without maize leaves, the positive priming effect was 7.6% for the bare fallow soil and 2.7% for the old-field soil from Hailun station. For soils from Shenyang station, the negative priming effect was 8.2% for the bare fallow soil and 4.1% for the old-field soil (Fig 7). Across all treatments and through the entire experimental period, approximately 9.8% of the initial SOC was mineralized into CO_2_ for soils from Hailun station, and 16.5% for soils from Shenyang station (S2 Table).

### The dynamics of soil microbial biomass carbon

**Fig 8:**
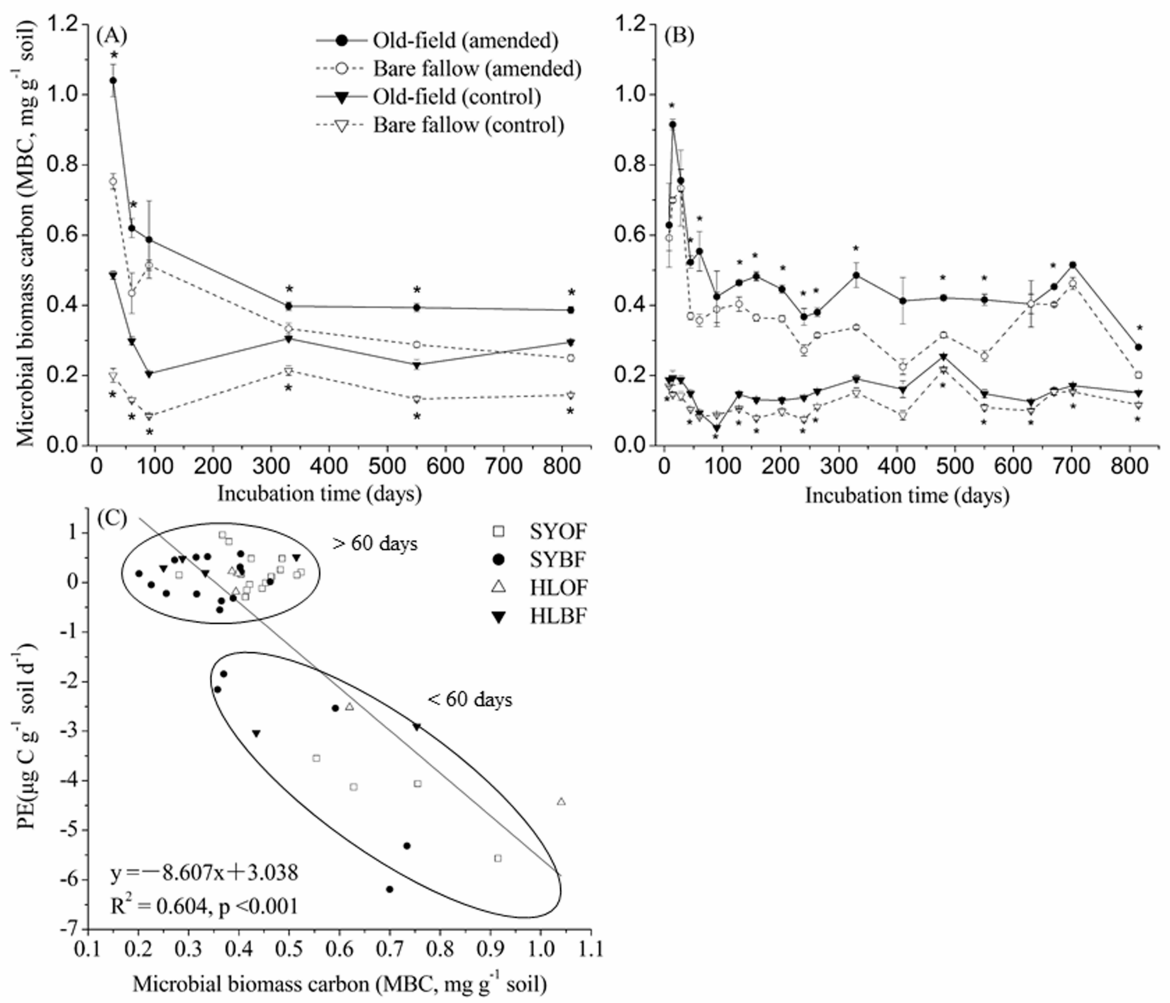
**Soil microbial biomass carbon (mg g**^**-1**^ **soil) from Hailun (A) and Shenyang (B) station through incubation time and correlation between PE (priming effect) and MBC (microbial biomass carbon) (C).** *indicates significant differences between old-field and bare fallow treatments. The PE data were the instantaneous priming effect. Both the PE and MBC data were mean values of 3 replicates at each sampling time (day-8, 14, 28, 45, 60, 90, 128, 158, 202, 240, 263, 330, 410, 480, 550, 630, 670, 702 and 815). Solid line is the regression line between PE and MBC. Error bars show ±1 SE.

For all soils and treatments, total microbial biomass carbon declined quickly during the initial 100 days of incubation, and stabilized afterwards (Fig 8A and 8B). There was a detectable period of increase in microbial biomass C in soils from Shenyang Station at later period (630–702 days), and the degree of this increase was higher for the bare fallow soil than the old-field soil. The addition of maize leaves significantly increased the total soil microbial biomass carbon for all the four soils; and the degree of increase was generally higher in bare fallow soils than in old-field soils from both Hailun Station and Shenyang Station during incubation (Fig 8A and 8B). On average, the addition of maize leaves caused 209.3% increase in total soil microbial biomass C in the bare fallow soil and 89.8% increase in the old-field soil from Hailun Station. For soils from Shenyang Station, the increase in total soil microbial biomass C due to the addition of maize leaves was 238.9% for the bare fallow soil and 245.8% for the old-field soil. We also found a significant negative correlation between priming effect (instantaneous rate) and the microbial biomass carbon (R^2^ = 0.604, P< 0.001, Fig 8C). The correlation between priming effect and microbial biomass carbon could be clearly divided into two phases: (1) During the initial 60-days, the priming effect was negatively correlated with the microbial biomass carbon; (2) From day-90 to the end of incubation, the priming effect was independent of the microbial biomass carbon.

## Discussion

We obtained quantitative priming effects of added maize leaves on SOC decomposition under treatment factors of two soil types (Mollisol from Hailun and Alfisol from Shenyang), two treatments of SOC stabilities (bare fallow representing stable SOC and old-field representing labile SOC) within each soil types, and at many different times of measurements throughout 815 days of incubation. Soil type (p<0.0001) and incubation time (p<0.0001) significantly influenced the priming effect, but not SOC stability (p = 0.886) (S1 Table). However, all levels of interactions (Incubation time, SOC Stability and soil type) were statistically significant for controlling the priming effect (S1 Table). This was due to the influence of SOC stability on the priming effect was overwhelmed by the measurement times and by soil types. Therefore, the following discussion first focuses on the dynamic changes of the priming effect through time, then on the interactive nature of the priming effect with respects to SOC stability, soil type, and incubation time.

### The dynamics of the priming effect

The pattern of changes in the instantaneous priming effect (Fig 5) and in the cumulative priming effect (Fig 6) demonstrates three distinctive phases for all soils: (1) a strong negative priming phase during the first period (≈ 0–90 days); (2) a pulse of positive priming (≈ 70–160 days for soils from Hailun station, ≈ 140–350 days for soils from Shenyang station); and (3) a relatively stabilized phase of priming at a lower level during the last stage of the incubation. The amount of primed SOC per unit of microbial biomass carbon indicates a similar pattern (S3 Fig). The first phase coincides with the initial fast decline in decomposition rates of added maize leaves (S2 Fig) and of SOC in the soils without maize leaves (Fig 2). The first phase is likely a microbial response to the pulse of water soluble substrates released either as a result of soil disturbance in the case of soil incubation without maize leaves or as a direct result of added fresh substrates in the case of maize leaf addition. Microbial assimilation of the available substrates and associated growth of microbial biomass should dominate the first phase, causing the phenomenon of negative priming, possibly because the hypothesized “preferential substrate utilization” by which soil microbes switch to added substrates and use less of original substrates [32]. The negative correlation between the priming effect and microbial biomass carbon during the initial 60-days (Fig 8C) indeed supports this hypothesis. This phenomenon has been implicated in the review by Blagodatskaya & Kuzyakov [23] in which they shown that the amount of added fresh substrates beyond 50% of microbial biomass C was negatively correlated with the priming effect. Furthermore, negative rhizosphere priming effects tend to also occur during early periods of studies [33]. However, the first phase of fast changing dynamics may not always result in a negative priming effect, on the contrary, many positive priming effects during this phase have been reported across various quantities and qualities of added substrates and with different lengths of experimental durations [23, 34]. It is likely that the potentially complex interactions between microbial utilization of “native substrates” from soil disturbance and the utilization of the added substrates play a pivotal role in determining the directions of priming effects during the first phase [9].

The second phase in this dynamic pattern is featured with a pulse of positive priming effects in all soils (Figs 5 and 6), and coincides with the second peak in decomposition rates of the added maize leaves (S2 Fig). There are several potential mechanisms behind this phenomenon. Based on the microbial community succession hypothesis (e.g. from r- to K-strategy) [35], the second phase should be dominated by microbes with K-strategy such as specialized bacteria and fungi which have been shown to be responsible for positive priming effects [36]. It is also likely that the C (and N) assimilated into microbial biomass during the first phase gets mineralized during the second phase due to the increased turnover of microbial biomass due to the die-out after the initial pulse of growth induced by the fresh substrate input [16, 32]. Indeed, the second phase occurred during the transition period from relatively high microbial biomass C to a stabilized lower microbial biomass, indicating a faster microbial turnover rate (Fig 8C). Furthermore, enhanced release of extracellular enzymes due to the increase in total soil microbial biomass and activities can also be responsible for the pulse of positive priming during the second phase (Fig 8) [19]. All three mechanisms are not necessarily mutually exclusive, on the contrary, they possibly operate in consort. This is implicitly supported by the fact that the second phase coincides with the second peak in decomposition rates of the added maize leaf C (Fig 5, S2 Fig) which might have resulted from microbial community succession (i.e., from r- to K-strategies), accelerated microbial turnover, and a boosted extracellular enzyme release all together. Further illumination on these possibilities is highly needed.

The third phase in the dynamic pattern of the priming effect is characterized with persistent priming at a low level from approximately day-300 onward (Fig 5). This phase of priming tends to corroborate with a stabilized microbial biomass level after a faster turnover period (Fig 8). This phenomenon has been noted in a study using straws as the fresh SOC input and with an experimental duration of 209 days [7]. Results from a meta-analysis have also indicated the existence of this phenomenon, even though only a few data points came from studies with experimental durations longer than 150 days [9]. Most published studies on the priming effect have focused on short-term outcomes, and largely overlooked this longer-term but persistent priming effect [6, 9]. This phase of persistent priming at a longer-term but at a lower level has important implications. If the priming of fresh input on the decomposition of older SOC can generally persist beyond the annual time scale, input-driven dynamics become a crucial component in the cycling of stabilized SOC which is the main soil C stock globally [1], therefore, incorporating the priming effect at different time scales into global C models is highly desirable [9]. This persistent priming effect can also act as a negative feedback mechanism behind the carbon saturation hypothesis [37] which stipulates that increased fresh SOC input may not further result in a noticeable increase in soil carbon storage once the maximum soil carbon stabilization/protection capacity has been reached as increases in fresh input may induce more priming effect. This mechanism has been implicitly demonstrated in a study using long-term soil carbon data and model-fitting approaches [21]. However, more empirical evidence supporting the general significance of this longer-term persistent priming effect is needed in the future.

Putting all three phases together, our results highlight the importance of temporal scales in studying the priming effect. Short-term determinations of overall (or cumulative) priming effects under particular environmental settings do not necessarily provide the whole picture. Many published reports have often been limited to the first phase which tend to be highly uncertain in terms of the direction (i.e., positive or negative), the nature (apparent or real priming) and the magnitude of the priming effects being reported [9, 34]. Therefore, longer temporal scales coupled with determination of dynamic indexes such as changes of active microbial composition, turnover of active microbial biomass, and changes in microbial metabolisms are recommended for future studies.

### SOC stability and soil type effects on priming

Overall, our results showed a weak effect of SOC stability on priming effects (S1 Table). During the first phase in the dynamic pattern of the priming effect, the priming effect was 21.4% more negative for the labile SOC in the old-field soils than for the stable SOC in bare fallow soils (Fig 5). However, this difference disappeared at the end of the second phase (Figs 5 and 6). Whereas, during the entire third phase, the positive priming effect was 48% higher for the stable SOC than for the labile SOC. The weaker positive priming for the sable SOC in the two bare fallow soils during the second phase was reasonable as 23 years without fresh organic input would induce the soil microbial community to a greater level of “starvation” than the old-field soils. Consequently, the decomposer communities would assimilate more SOC, leading to less positive priming during the second phase [32]. The slightly more positive priming effect for the stable SOC in the bare fallow soil during the third phase might have resulted from the higher increase of total microbial biomass C in the bare fallow soil than in the old-field soil (232% vs. 208% on average) (Fig 8). Overall, the influence of SOC stability on the priming effect is relatively weak, and therefore, we conclude that stable SOC (as in the case of the bare fallow soil) can be primed to a roughly similar level as labile SOC (as in the case of the old-field soil) in accordance with the result from a published study [7].

The influence of soil type on the priming effect was apparently higher than SOC stabilities (S1 Table). Compared to the soils from Shenyang station, the soils from Hailun station showed a similar negative priming effect during the first phase, a bigger peak of positive priming effect during the second phase, a consistently higher positive priming effect during the third phase in the overall dynamic pattern of the priming effect in the entire experiment (Figs 5 and 6). Reflecting this overall dynamic pattern, the cumulative priming effect at the end of the experiment (day-815) was significantly positive for soils from Hailun station, but significantly negative for soils from Shenyang station (Fig 7). These differences in the priming effect between soils from Hailun and Shenyang largely stem from the differences in soil properties (Table 1). Salomé et al (2010) has shown that higher content of less mineralizable SOC tended to produce higher priming effect [15]. Soil texture is widely known as one of the most important soil properties in controlling SOC dynamics [21, 38–40], especially considering that SOC can become physically protected from decomposition by clay particles [41–42]. The soils from Hailun have higher organic C and N content and higher clay content than the soils from Shenyang. The higher amount of added maize leaves remaining in soils from Hailun than soils from Shenyang (21% vs. 17%) at the end of the experiment (S2 Table) indeed indicated higher level of clay protection in soils form Hailun. Therefore, the greater clay content in soils from Hailun station can be the main reason for its relatively lower mineralizability than soils from Shenyang station. Relatively higher priming effect for soils with higher clay-protected SOC has been reported in the published literature [4, 17, 39].

## Conclusions

Overall the priming effect of added maize leaves on SOC decomposition varied widely during the entire experiment. The variation of the priming effect through time seemed to show three distinctive phases for all soils: (1) a strong negative priming phase during the first period; (2) a pulse of positive priming phase in the middle; and (3) a relatively stabilized phase of priming during the last stage of the incubation. The influences of soil types and SOC stabilities on the priming effect could only be understood when the three distinctive phases were analyzed separately.

Due to differences in soil properties, the two soil types produced different cumulative priming effects at the end of the experiment, a positive priming effect of 3–7% for the Mollisol from Hailun station and a negative priming effect of 4–8% for the Alfisol from Shenyang station. Although soil types and incubation times modulated most of the variability of the priming effect, relative SOC stabilities also influenced the priming effect for a particular soil type and at a particular dynamic phase. The stable SOC from the bare fallow treatment tended to produce a narrower variability during the first phase of negative priming and during the second phase of positive priming. Averaged over the entire experiment, the stable SOC (i.e., the bare fallow) was at least as responsive to priming as the relatively labile SOC (i.e., the old-field) if not more responsive.

This study demonstrates the three distinctive phases of the priming effect as the duration of our experiment was much longer than published reports. To the best of our knowledge, our current study is among the first that measured priming effects up to 815 days after fresh substrate addition. We overcome the commonly known logistic limitations by using an improved measurement system and a large soil sample size. Our results highlight the importance of studying the priming effect by investigating the temporal dynamics over the annual time scale.

## Supporting information

S1 Fig The geographical locations of Hailun (47°26′N, 126°38′E) and Shenyang (41°32′N, 122°23′E) agricultural experimental stations. (TIF)

S2 Fig CO_2_ efflux rates (mg CO_2_-C g^-1^ maize leaf C remaining d^-1^) from the mineralization of maize leaves in the four soils (HLOF: Old-field soil from Hailun; HLBF: Bare fallow soil from Hailun; SYOF: Old-field soil from Shenyang; SYBF: Bare fallow soil from Shenyang). The inserts show the CO_2_ efflux from day-45 to day-815. Error bars show ±1 SE.
(TIF)

S3 Fig The amount of primed carbon per unit mass of microbial biomass carbon (mg C g^-1^ MBC) in the four soils (HLOF: Old-field soil from Hailun; HLBF: Bare fallow soil from Hailun; SYOF: Old-field soil from Shenyang; SYBF: Bare fallow soil from Shenyang). Error bars show ±1 SE. The dynamic change of the microbial biomass specific priming over the entire experimental duration shows three distinctive phases: (1) negative priming but rapidly increasing phase (≈ 0–90 days), (2) a pulse of positive priming (≈ 90–410 days), and (3) stabilized phase (> 410 days). Error bars show ±1 SE.
(TIF)

S1 Table Analysis of variance (ANOVA) table for instantaneous priming effect with three main factors (SOC stability: Labile (old-field), stable (bare fallow); soil type: Mollisol (Hailun Station), Alfisol (Shenyang Station); incubation time: 26 times). Instantaneous priming effect was calculated by subtracting the CO_2_ efflux rate of the soil only control from the SOC-derived CO_2_ efflux rate of the treatment with maize leaves at the same sampling time for each soil type.
(DOCX)

S2 Table Soil carbon balance of old-field and bare fallow soils at the end of incubation (mg C g^-1^ soil). Each number in parentheses is a standard error for the mean in the front.
(DOCX)
