# Supplementary figures and images for: Priming effects on labile and stable soil organic carbon decomposition: Pulse dynamics over two years
Source: PLoS One. 2017 Sep 21;12(9):e0184978. doi: 10.1371/journal.pone.0184978 (PMC5608328; doi:10.1371/journal.pone.0184978)

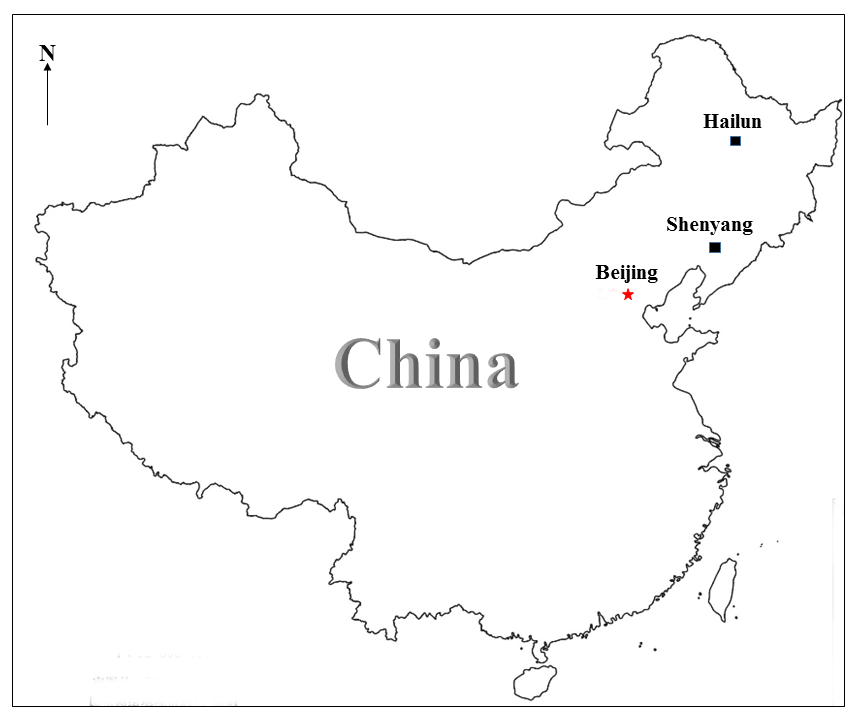

Supplement: S1 Fig — (TIF) [file pone.0184978.s001.tif]
