# Supplementary material for: Priming effects on labile and stable soil organic carbon decomposition: Pulse dynamics over two years
Source: PLoS One. 2017 Sep 21;12(9):e0184978. doi: 10.1371/journal.pone.0184978 (PMC5608328; doi:10.1371/journal.pone.0184978)
Supplement: S1 Table — Instantaneous priming effect was calculated by subtracting the CO2 efflux rate of the soil only control from the SOC-derived CO2 efflux rate of the treatment with maize leaves at the same sampling time for each soil type. (DOCX) [file pone.0184978.s004.docx]

**S1 Table. Analysis of variance (ANOVA) table for instantaneous priming effect with three main factors (SOC stability: labile (old-field), stable (bare fallow); soil type: Mollisol (Hailun Station), Alfisol (Shenyang Station); incubation time: 26 times).** Instantaneous priming effect was calculated by subtracting the CO_2_ efflux rate of the soil only control from the SOC-derived CO_2_ efflux rate of the treatment with maize leaves at the same sampling time for each soil type.

| **Source** | **df** | **SS** | **MS** | **MS (% total)** | **F** | **P** |
| --- | --- | --- | --- | --- | --- | --- |
| **Incubation time (A)** | 25 | 1183.01 | 47.32 | 65.48 | 96.46 | <0.0001 |
| **SOC stability (B)** | 1 | 0.01 | 0.01 | 0.01 | 0.02 | 0.886 |
| **Soil type (C)** | 1 | 14.61 | 14.61 | 20.22 | 29.78 | <0.0001 |
| **A × B** | 25 | 69.53 | 2.78 | 3.85 | 5.67 | <0.0001 |
| **A × C** | 25 | 44.77 | 1.79 | 2.48 | 3.65 | <0.0001 |
| **B × C** | 1 | 2.05 | 2.05 | 2.84 | 4.18 | 0.042 |
| **A × B × C** | 25 | 80.20 | 3.21 | 4.44 | 6.54 | <0.0001 |
| **Residual** | 312 | 153.06 | 0.49 | 0.68 |  |  |
