# Supplementary material for: Priming effects on labile and stable soil organic carbon decomposition: Pulse dynamics over two years
Source: PLoS One. 2017 Sep 21;12(9):e0184978. doi: 10.1371/journal.pone.0184978 (PMC5608328; doi:10.1371/journal.pone.0184978)
Supplement: S2 Table — Each number in parentheses is a standard error for the mean in the front. (DOCX) [file pone.0184978.s005.docx]

**S2 Table. Soil carbon balance of old-field and bare fallow soils at the end of incubation (mg C g^-1^ soil).** Each number in parentheses is a standard error for the mean in the front.

|  | Hailun old-field | | Hailun bare fallow | | Shenyang old-field | | Shenyang bare fallow | |
| --- | --- | --- | --- | --- | --- | --- | --- | --- |
|  | amended | control | amended | control | amended | control | amended | control |
| Initial SOC | 35.55 | 35.55 | 21.81 | 21.81 | 16.60 | 16.60 | 11.44 | 11.44 |
| Maize C | 11.37 | 0 | 11.37 | 0 | 11.37 | 0 | 11.37 | 0 |
| Total C start | 46.92 | 35.55 | 33.18 | 21.81 | 27.97 | 16.60 | 22.81 | 11.44 |
| Total CO_2_ | 12.25(0.15) | 3.33(0.07) | 11.36(0.41) | 2.11(0.03) | 12.07(0.12) | 2.83(0.04) | 11.26(0.36) | 1.95(0.03) |
| Maize-CO_2_ | 8.83(0.13) | － | 9.09(0.33) | － | 9.35(0.11) | － | 9.49(0.32) | － |
| SOC-CO_2_ | 3.42(0.04) | 3.33(0.07) | 2.27(0.08) | 2.11(0.03) | 2.72(0.02) | 2.83(0.04) | 1.77(0.05) | 1.95(0.03) |
| Total C end | 34.67 | 32.22 | 21.82 | 19.70 | 15.90 | 13.77 | 11.55 | 9.49 |
| % SOC loss | 9.62 | 9.37 | 10.41 | 9.67 | 16.39 | 17.05 | 15.47 | 17.05 |
| % C_4_ loss | 77.66 |  | 79.95 |  | 82.23 |  | 83.47 |  |
